# Validation of a Laboratory-Developed Triplex Molecular Assay for Simultaneous Detection of Gastrointestinal Adenovirus and Rotavirus in Stool Specimens

**DOI:** 10.3390/pathogens9050326

**Published:** 2020-04-27

**Authors:** Rachel R. Higgins, Adriana Peci, Mark Cardona, Jonathan B. Gubbay

**Affiliations:** 1 Public Health Ontario Laboratory, 661 University Avenue, Toronto, ON M5G 1M1, Canada; adriana.peci@oahpp.ca (A.P.); mark.cardona@oahpp.ca (M.C.); jonathan.gubbay@oahpp.ca (J.B.G.); 2 Laboratory Medicine and Pathobiology, University of Toronto, Toronto, ON M5S 2V8, Canada; 3 The Hospital for Sick Children, 555 University Avenue, Toronto, ON M5G 1X8, Canada

**Keywords:** multiplex, enteric, gastrointestinal, adenovirus, rotavirus, validation

## Abstract

Enteric viral pathogens causing gastroenteritis include adenovirus and rotavirus, among others. Rotavirus is the leading cause of severe diarrhea in infants and young children worldwide. Among the adenoviruses known to cause gastroenteritis are those of species F (serotypes 40, 41). Here, we describe the development and validation of a laboratory-developed gastrointestinal triplex rRT-PCR (triplex) assay that targets adenovirus and rotavirus. Stool specimens were tested from patients across Ontario. Specimens were previously tested for adenovirus and/or rotavirus by electron microscopy (EM) or immunochromatographic test (ICT). Triplex sensitivity, specificity, positive and negative predictive values compared to Seegene assay (a commercial assay used here as the standard reference method) were 100%, 97.8%, 86.0%, 100% for adenovirus, and 99.1%, 98.4%, 96.3%. 99.6% for rotavirus, respectively. The triplex assay had a 95.2% and 97.3% overall percent agreements (OPAs) when compared to EM for adenovirus or rotavirus detection, respectively, and an OPA of 90.9% when compared to rotavirus ICT for rotavirus detection. Triplex assay exhibited similar performance to the Seegene assay for both adenovirus and rotavirus and detected more adenovirus and rotavirus than traditional testing methods. The high performance along with lower cost and reduced turnaround time makes the triplex assay a desirable testing method for a clinical microbiology laboratory.

## 1. Introduction

Infectious diarrhea is the second most common infectious condition worldwide, after acute respiratory infections, affecting humans of all age groups, but mostly the young, the elderly, the immunocompromised patients and people in enclosed communities such as hospitals, nursing homes, military bases and cruise ships [1,2,3]. Known enteric viral pathogens, as causative agents of gastroenteritis, include adenovirus, rotavirus, norovirus, sapovirus and astrovirus [4,5,6]. Several bacterial pathogens, such as Campylobacter jejuni, enterotoxigenic Escherichia Coli, Shigella species and Vibrio cholera, are also reported to cause gastroenteritis, particularly in developing countries and refugee camps [7]. Viral gastroenteritis is reported to be more common than bacterial gastroenteritis; however, due to lack of molecular testing in developing countries, it is difficult to quantify it properly [7].

Among viruses, norovirus is the most common cause of acute gastroenteritis outbreaks in humans [8,9]. Rotavirus was the leading pathogen causing diarrhea in infants and young children in the United States, before the introduction of vaccine in 2006. Rotavirus infection in adults and the elderly is less likely; however, when it happens, a prolonged and severe infection can occur, particularly among immunocompromised patients [10].

Since the introduction of publicly funded rotavirus vaccine in Ontario, in August 2011, the prevalence of rotavirus has been reduced [6,11]. Stool specimens showed a significant decrease in rotavirus percent positivity from 14.4% to a 36-month period before implementation of vaccine to 6.1% during a 46-month period after rotavirus vaccine implementation [11]. 

Of the adenoviruses reported to cause gastroenteritis are those of species F (serotypes 40 and 41) [12,13]. Adenovirus infection can be transmitted via aerosolized droplets or via fecal oral spread, or direct conjunctival inoculation including contact with tap water, and environmental surfaces [13]. Symptoms most frequently reported include watery diarrhea, vomiting, and low temperature [13]. While adenovirus is less common than rotavirus, the duration of diarrhea is longer [12]. Adenoviruses can be infectious even after several weeks in moisture free environments and the virus is resistant to many disinfectants because it is a non-enveloped DNA virus [14]. Viral persistence of adenovirus in the gastrointestinal tract and shedding for a long period are common and frequently reported to not be associated with clinical symptoms [15]. 

The great burden of viral gastroenteritis on health care due to the related illness and hospitalization, particularly among younger and older age groups, highlights the need for fast, sensitive and reliable diagnostic assays to guide infection control measures. The existing methods for detecting enteric viruses at Public Health Ontario (PHO) laboratory rely on conventional and labor-intensive electron microscopy (EM), which has low sensitivity, is subjective, and it requires highly skilled and experienced personnel. Imunochromatographic test (ICT) is another method used at PHO laboratory, which is rapid and simple to use but it lacks sensitivity and specificity [16]. At PHO laboratory virus culture is used to detect enteroviruses only during the summer-fall months.

Real Time Polymerase Chain Reaction (RT-PCR) assays have been successfully implemented for the detection of adenovirus [17] and/or rotavirus [18]. The simultaneous detection of several viruses can be accelerated and simplified by the use of a multiplex RT-PCR assay. Commercial methods for detection of both viruses are available but are expensive and not amenable to multiplexing of additional viruses [19]. 

The objective of this study was to develop an in-house reverse transcription RT-PCR (rRT-PCR) assay (triplex) that targets two diarrheal pathogens, adenovirus and rotavirus, along with an MS2 internal control. The triplex assay was developed to replace the two conventional assay methods (EM and rotavirus ICT) and complement the norovirus rRT-PCR assay in use at PHO laboratory that targets norovirus GI and GII, the two most common pathogenic genotypes in humans. In addition, the triplex assay will be essential when virus titer is low and high sensitivity is desired, as in the case of water contamination. Here, we describe the development and validation of the triplex assay and its evaluation against Seegene, EM and rotavirus ICT. Knowledge gained from this study can be used to expand this laboratory-developed multiplex assay to simultaneously detect additional viral pathogens causing gastroenteritis. 

## 2. Materials and Methods

**Figure 1:**
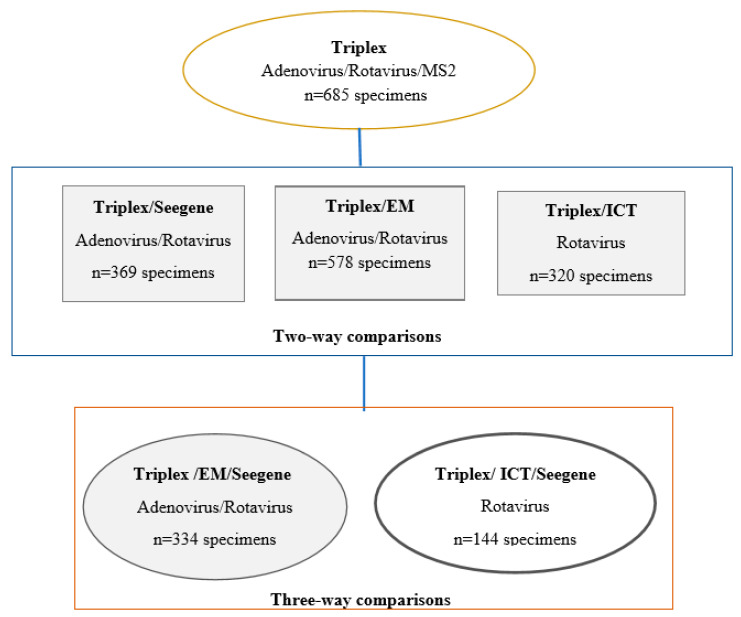
The distribution of specimens used for performance evaluation of triplex Adenovirus/Rotavirus assay against Seegene, Electron Microscopy and Imunochromatographic test.

This study utilized clinical stool specimens submitted to PHO laboratory for routine gastrointestinal virus testing, including adenovirus and rotavirus, from patients across Ontario. Of these, a convenience subset (n = 685), consisting of available positive and negative specimens for rotavirus and/or adenovirus was used for this study (Figure 1). These specimens were submitted to PHO laboratory over a 4 year period, between February 2009 and February 2013. For the purpose of this validation, positive specimens for adenovirus or rotavirus refer to those previously confirmed positive by EM and/or ICT tests.

A panel of controls as illustrated in Table 1, comprised of positive bacterial specimens, viral specimens and bacterial culture isolates was used to determine assay specificity.

### 2.1. Testing Methods

All specimens were previously tested for adenovirus or rotavirus by EM (EM, Philips CM10 or Field Electronic and Ion Company (FEI) Morgagni 268, Eindhoven, The Netherlands) or for rotavirus only by rotavirus ICT, with commercial rotavirus antigen detection kits (RIDAQUICK Rotavirus (Phoenix Airmid Biomedical Corp., Oakville, ON, Canada), or Rotascreen (Microgen Bioproducts Ltd., Camberley, UK)). 

The triplex assay developed for the detection of rotavirus/adenovirus/MS2 and evaluated here, is an in-house laboratory-developed multiplex rRT-PCR viral diarrheal assay. This triplex assay consists of rRT-PCR on the ABI 7500 fast instrument (Applied Biosystems, CA, USA). For the purpose of this validation, a group of specimens included in this study was also tested for rotavirus and adenovirus using the Seegene, (Seeplex Diarrhea-V ACE Seegene, Seoul, Korea) commercial assay [20]. Triplex assay results were evaluated against Seegene assay, used here as the reference standard method and against EM and rotavirus ICT, two additional “non-standard” reference methods.

### 2.2. RNA and DNA Extraction

All specimens used in this study were stored at −70 °C prior to thawing and testing. Stool specimens were homogenized (20% weight/volume) in sterile water, centrifuged at 3000× *g* for 20 min at room temperature and 250 uL of each clarified supernatant was spiked with the MS2 control (Roche, Mannheime, Germany) at 1 × 10^4^ copies per specimen and subjected to automated nucleic acid extraction. Total nucleic acid was eluted in a final volume of 110 uL, of which 5 uL was used for PCR amplification. Nucleic acid was extracted directly from diluted stool specimens using (NucliSENS easyMag, bioMérieux, France) as recommended by the manufacturer [21]. Bacterial cultures grown in appropriate culture media at PHO laboratory were diluted to 0.5 McFarland turbidity standard in phosphate buffer saline (Gibco PBS, Grand Island, NY, USA) prior to automated extraction on the NucliSENS easyMAG.

The adequacy of nucleic acid extraction was confirmed by RT-PCR amplification of human GAPDH gene and/or *Bacterioidis fragilis* (*B. fragilis*) strain VPI 2553 (ATCC 25285). *GAPDH* was used in addition to *B. fragilis* in a subset of infant’s specimens (n = 140) since *B. fragilis* may not be present in breast-fed infants’ stool due to maternal protection [22,23]. The remaining specimens (n = 545) were tested by *B. fragilis* only.

### 2.3. PCR Amplification

Amplification and detection of nucleic acid using the triplex rRT-PCR gastrointestinal virus assay was carried out using the ABI7500 RT-PCR instrument. The rRT-PCR amplification was performed using Qiagen one step RT-PCR kit (Qiagen, Frederick, MD, USA). Primers and probes used in the triplex assay are shown in Table 2 [17,18,24,25].

Primers and probes were chosen from the most conserved regions for both viruses; 103 bp in the hexon gene region for adenovirus and non-structural protein 3 (NSP3) gene was used for rotavirus. These together with the use of degenerate primers and probes allowed detection of all serotypes including A, B, C, D, E, F, G serotypes for adenovirus and A, B, C, D, E, F, G, H, I, J for rotavirus [10,12].

The rRT-PCR reaction conditions were as follows: Five microliters (uL) of extracted DNA or RNA template was used in the triplex assay in a final volume of 25 uL per reaction. The final concentrations of primers and probes in the reaction were 300 nM and 100 nM for primers and dual-labelled probes, respectively. MS2 primer and probe concentrations were 100 nM and 50 nM, respectively. The assay was performed in 25 uL reaction volume containing 1× Qiagen one step RT-PCR kit. Cycling conditions were as follows: One cycle of reverse transcription of RNA at 48 °C for 30 min, one cycle of activation/denaturation step at 95 °C for 15 min followed by 40 cycles of amplification, each with 15 s denaturation at 95 °C and 60 s amplification/extension at 60 °C. Analysis of amplification curves utilized threshold 3 to 15 with manual adjustments of base line. Amplification of specimens for the Seegene assay was performed on Bio-Rad thermocyler (Bio-Rad Laboratories, Hercules, CA, USA) according to the manufacturer’s product insert. Since rotavirus is an RNA virus and adenovirus a DNA virus, several components of reaction conditions were tested and optimized including primer/probe concentrations, PCR cycling conditions, annealing temperature etc. Singleplexes of adenovirus and rotavirus were initially developed and performance was compared to duplex assay (adenovirus/MS2 and rotavirus/MS2) and later to triplex assay where all three targets were included (adenovirus/rotavirus/MS2). rRT-PCR inhibitory effects in the triplex assay were assessed by co-amplification of an internal MS2 control included in the primer-probe mix. In the event of PCR failure, the nucleic acid was re-extracted from stool specimens and triplex PCR assay was repeated. The MS2 was used as an internal control to eliminate false negatives results due to assay failure. MS2 was calculated such that at the applied concentration (10^−4^ ug/mL), mean CT value of 22.5, standard deviation ±1.034, and range 22–24 were obtained. The assay developed is qualitative, and amplification of MS2 in the absence of target pathogens was consistently maintained. In the presence of target pathogen, the CT value for MS2 increased based on the viral titer. The higher the concentration of target viruses, the higher the CT value for MS2. 

### 2.4. Accuracy

Clinical specimens previously tested for adenovirus and/or rotavirus were used for this study. Controls included during test validation were excluded from the performance evaluation analyses. Results of triplex rotavirus/adenovirus were compared with Seegene, the standard reference method in two-way performance comparison analysis. Performance indicators such as sensitivity, specificity, positive predicted value (PPV) and negative predictive value (NPV) were calculated with 95% confidence intervals. Results of triplex assay testing for both adenovirus and rotavirus were further compared to EM, and for rotavirus only, to rotavirus ICT, two non-standard reference methods. Positive percent agreement (PPA), negative percent agreement (NPA) and overall percent agreement (OPA) were reported when compared with the non-reference standard methods, as recommended by FDA guidelines for evaluating diagnostic tests [26]. Chi-square was also employed to compare results between two methods (triplex versus Seegene; triplex versus EM; and triplex versus ICT). Three-way comparisons were also performed on specimens tested for adenovirus and rotavirus by triplex, EM and Seegene; and for rotavirus only by triplex, rotavirus ICT, and Seegene. In both comparisons, Seegene was used as the standard reference method. Three-way comparisons were used to increase the accuracy of this validation and to reduce the effect of discrepant results between methods [26].

### 2.5. Analytical Sensitivity: Limit of Detection

The 95% limit of detection (LOD) in copies per reaction for each virus in the multiplex rRT-PCR gastrointestinal assay was determined using commercially produced plasmid DNA (Seegene, South Korea) and/or quantified tissue culture fluid (TCF, Zeptometrix Corporation, NY, USA). TCF for each viral target was serially diluted 10-fold to 10^−12^ of the starting material in PCR grade water and tested in the triplex assay. The respective 95% LODs were assessed by testing a minimum of four replicates of each of the 12 dilution steps. The LOD indicates the lowest concentration of genome target at which a pathogen will be detected with a probability of 95%. The LODs and 95% confidence interval for each target was determined using probit regression (SPSS software regression package version 18.0, SPSS, Chicago, IL, USA).

### 2.6. Precision: Repeatability (Intra Assay Precision) and Reproducibility (Inter Assay Precision)

Intra and inter assay precision was assessed by using data generated from the LOD calculation and from additional testing of specimens on three different days. Concordance for intra and inter-assay reproducibility were calculated.

### 2.7. Turnaround Time

Turnaround time (TAT) was calculated for the triplex assay based on five runs, each run of 96 specimens completed by one laboratory staff member. TAT reflected the average time to process 96 specimens, including hands-on time, assay time and resulting/reporting times (pre-analytical, analytical and post-analytical components). 

### 2.8. Analytical Specificity

The absence of cross reactivity with other unrelated pathogens was assessed by a panel of 32 control specimens comprised of primary specimens as well as bacteria and culture isolates of known pathogens. Interference was assessed to show that the presence of other pathogens did not interfere with virus detection. 

### 2.9. Ethics Statement

This study was considered exempt from Public Health Ontario’s Research Ethics Board review as it involved de-identified stool specimens that were tested as part of a routine clinical virology services provided by PHO laboratory. 

## 3. Results

This study employed a convenience selection of previously tested specimens for gastrointestinal viruses. Specimens were submitted as part of routine clinical testing by clinicians across Ontario, Canada to PHO laboratory. Among 685 unique specimens included in this study 349 (50.9%) and 39 (5.7%) were positive for rotavirus and adenovirus, respectively by traditional testing methods (EM and/or ICT) (Table 1). The median age of patients for which age was reported (n = 583) was 1 year old with a range of 2 months to 97 years. Of 562 patient’s specimens that had gender reported 42.3% (238/562) were female and 57.6% (324/562) were male.

## 4. Accuracy

### 4.1. Performance Characteristics of Triplex Assay Compared to Seegene PCR Assay 

Due to limited resources, only 369 unique specimens from samples included in this study were tested by both triplex and Seegene assays for detection of adenovirus and rotavirus as illustrated in (Figure 1 and Table 3A). For this comparison a performance below 85% was considered as ‘below average’, at 86–90% as ‘average’ and above 90% as ‘above average’. Sensitivity, specificity, PPV and NPV for adenovirus were 100%, 97.8%, 86% and 100%, respectively and for rotavirus were 99.1%, 98.4%, 96.3% and 99.6%, respectively (Table 4A).

### 4.2. Performance Characteristics of Triplex Assay Against EM or Rotavirus ICT (Two Non-Standard Reference Testing Methods)

Five hundred and seventy-eight specimens had both triplex assay and EM results for adenovirus and rotavirus (Figure 1 and Table 3B). Three hundred and twenty specimens were tested by triplex assay and rotavirus ICT for detection of rotavirus (Table 3C). Performance indicators of triplex for the comparison with these non-standard reference testing methods (PPA, NPA and OPA) are reported in Table 4B for each paired comparison. Triplex assay had an OPA of 95.6% when compared to EM for both adenovirus and rotavirus and an OPA of 88.1% when compared to rotavirus ICT for rotavirus detection. Performance indicators of the triplex assay testing for adenovirus and rotavirus using Seegene as the standard reference method are reported in Table 4A.

### 4.3. Three-Way Comparison of Triplex Assay Against EM and/or ICT

To resolve discordant results, the gastrointestinal triplex assay is evaluated against EM and Seegene assay for adenovirus and rotavirus or against ICT and Seegene assay for rotavirus. 

### 4.4. Adenovirus (Triplex-EM-Seegene)

A total of 334 specimens were tested by triplex assay, EM and Seegene assay for the detection of adenovirus and rotavirus (Figure 1 and Table 5A,B). 

Among 40 positive adenovirus specimens by Seegene, 32 (80%) were confirmed by both triplex and EM. Subsequently, among 294 negative adenovirus specimens by Seegene, 286 (97.2%) were also confirmed by both EM and triplex. The overall percent agreement between triplex, EM and Seegene for adenovirus detection was (318/334) 95.2% (Table 5A).

### 4.5. Rotavirus (Triplex-EM-Seegene)

For rotavirus testing, percent agreement among positive rotavirus specimens by Seegene was 97% (97/100); percent agreement among negative rotavirus specimens by Seegene was 97.4% (228/234); and the overall percent agreement between triplex, EM and Seegene for detection of rotavirus was 97.3% (325/334) (Table 5B). 

### 4.6. Rotavirus (Triplex-ICT-Seegene)

A total of 144 specimens were tested by triplex assay, rotavirus ICT and Seegene assay for rotavirus detection (Figure 1 and Table 5C). Percent agreement among positive rotavirus specimen by Seegene was 88.5% (46/52); percent agreement among negative rotavirus specimens by Seegene was 92.4% (85/92); and the overall agreement between triplex, ICT and Seegene for detection of rotavirus was (90.9%) 131/144.

### 4.7. Analytical Sensitivity: Limit of Detection

**Figure 2:**
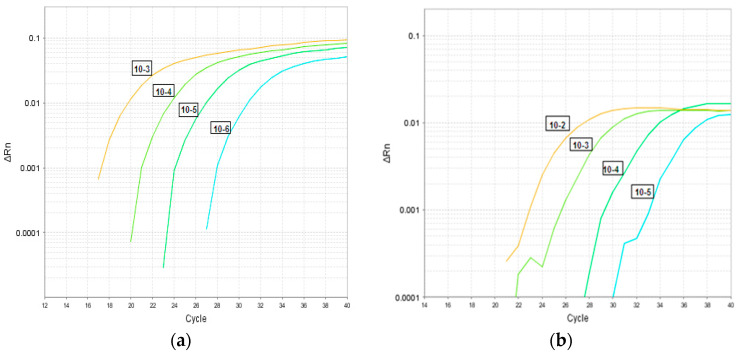
Limit of detection for the triplex assay for adenovirus (**a**) Adenovirus TCID_50_ = 10^−6.5^ and rotavirus (**b**) Rotavirus TCID_50_ = 10^−5.6^. **Footnote:** TCID_50_, Tissue Culture Infectivity Dose 50 is the median infectious dose in which 50% of the cells are infected with the virus.

Figure 2 depicts partial results (replicates not shown) of a typical LOD experiment using 10-fold serial dilutions of quantified TCF. As seen in Figure 2, adenovirus was detected at 10^−6.5^ TCID50/mL (Figure 2a) and rotavirus was detected at 10^−5.6^ TCID50/mL (Figure 2b). Using plasmid DNA, the 95% limit of detection in copies/reaction for adenovirus in the triplex assay was 0.45 copies or 30.8 genome equivalent and for rotavirus 4.5 copies or 10.2 genome equivalent. The estimated 95% LODs were 30.8 genome equivalent GEq/reaction for adenovirus and 10.2 GEq/reaction for rotavirus (results not shown).

### 4.8. Analytical Specificity

There was no cross-reaction with unrelated gastrointestinal bacteria and viruses in the analytical specificity experiments (Table 1).

### 4.9. Interference

Triplex primers and probes had no impact on assay sensitivity or performance for any individual target. The MS2, GAPDH and/or *B. fragilis* controls were detected in all clinical specimens. The presence of co-detection indicated the lack of interference when other pathogens were present in the specimen. Specifically, 12 (1.8%) specimens had rotavirus and adenovirus co-detected in the triplex assay. 

### 4.10. Precision and Turnaround Time

Intra and inter assay precision were both 100%. Average TATs for processing 96 specimens in one run were as follows: 9–10 h for the Seegene assay, 4–5 h for the triplex assay and 3 days for EM. 

## 5. Discussion

We describe the development and evaluation of a triplex molecular assay targeting adenovirus and rotavirus with MS2 included as an internal control. This is a laboratory developed multiplex rRT-PCR assay that simultaneously detects two gastroenteritis viral pathogens from clinical stool specimens. The incorporation of the MS2 internal control assessed for possible inhibition and prevents subsequent false negative reporting. Performance of triplex assay for rotavirus and adenovirus detection was compared with one standard and two non-standard reference testing methods. Three-way comparisons were also performed to enhance this validation and to help interpret discrepant results between methods [26]. 

Compared to Seegene, the triplex assay demonstrated above average sensitivity, specificity, and NPV for both adenovirus and rotavirus detections. Triplex assay performance was characterized by an above average PPV (96.3%) for rotavirus detection, but an average PPV for adenovirus detection (at 86%) (Table 4A). In comparison to Seegene assay, the triplex assay detected seven (16%) additional positive specimens for adenovirus and four (3.7%) additional specimens positive for rotavirus but missed one rotavirus positive specimen detected by the Seegene assay (Table 3A) (*p*-values < 0.001). The discrepancies between the two molecular methods could be explained by low viral loads found in these specimens, which plays a critical role in the assay sensitivity and test performance when comparing different methods [27]. Average sensitivities (89.3% and 86.2%) and above average specificities (99.9% and 97.2%) were reported in another study in which two commercial multiplex real time reverse transcription PCR assays were compared with a conventional RT-PCR assay, targeting adenovirus, rotavirus in addition to norovirus, human bocavirus and human paraechovirus [28]. Co-detection of both viruses was found among 12/685 (1.8%) of specimens tested by the triplex assay compared to 4/392 (0.7%) specimens tested by the Seegene assay. The four specimens detected in the Seegene assay were among the twelve co-detections identified by the triplex assay. This also suggests that the triplex assay had higher sensitivity for co-detecting co-pathogens compared to the Seegene assay (Table 3A and Table 4A), though false positivity is also a possibility.

When compared to EM, for detection of adenovirus and rotavirus, all PPA, NPA and OPA were above average (above 90%) (Table 4B). The triplex assay detected 23 (53%) additional adenovirus specimens and 20 (5%) additional rotavirus specimens compared to EM (Table 3B) (*p*-value < 0.001). These results are very similar to the observations of Logan at al., who reported 100% and 186% increase in adenovirus and rotavirus detection by rRT-PCR compared to EM [29]. Low sensitivity of EM compared to RT-PCR was reported previously, however, positive results by EM offer better correlation with disease in rotavirus infection [30]. PCR results cannot prove disease causality, due to the known presence of the virus shedding in asymptomatic individuals. Two and five stool specimens were positive by EM for adenovirus and rotavirus, respectively but failed to produce amplicons for adenovirus or rotavirus in the triplex assay (Table 3B). This has been reported before and it was suggested that specimens may contain virus particles that are morphologically similar to adenovirus or rotavirus but with little nucleotide sequence homology [31]. This could also be due to loss of virus particles caused by repeat freeze-thaw cycles. These specimens were likely tested by EM closer to disease onset and after prolonged incubation were tested retrospectively by the triplex assay.

In comparison to the rotavirus ICT method, the performance of the triplex assay for rotavirus detection was above average; PPA of (90.6%) and an average NPA of (85.6%) (Table 4B). The triplex assay detected rotavirus in 23 additional specimens compared to rotavirus ICT (Table 3C) (*p*-value < 0.001). The lower NPA was a result of 15 specimens in which rotavirus was detected by the rotavirus ICT test, but not by the triplex assay. This could be due to false positive results by the rotavirus ICT test, which are commonly reported for the rotavirus ICT test [32,33]. It could also be due to repeat freeze-thaw of specimens used for this validation study, which could cause genome degradation and lower detection. In fact, three of these specimens were confirmed to be rotavirus-positive by EM and one was also confirmed by the Seegene assay, suggesting genome degradation. 

When a new testing method is compared to a non-reference standard method, the results may be biased and the information provided for the new testing method may not be accurate [26]. Comparing triplex assay with EM performance for detection of both adenovirus and rotavirus, demonstrated above average OPAs of 95.6% for each comparison, respectively. Triplex assay performance for rotavirus detection compared to rotavirus ICT, shows an average OPA (88.1%). A three-way comparison using a standard-reference method enhances this validation and provide a better way of resolving discrepant results [26].

In summary, the triplex assay demonstrated similar performance to the Seegene assay for both adenovirus and rotavirus targets and performed better than traditional testing methods (EM and rotavirus ICT) used at PHO laboratory. The average PPA of the triplex assay detected for adenovirus in comparison to the Seegene assay could be due to mismatches with the viral templates and differences in oligonucleotide primers and probes used by these assay systems [33]. Possible explanations include higher sensitivity of the Seegene assay for adenovirus detection, or alternatively false positive results by Seegene. In addition to the high performance of the triplex assay, the high through-put, the lower cost compared to commercial assays and EM and the reduced TAT makes it a more desirable testing method. The findings of this study have been extended to guide the planned development of a multiplex platform to test stool specimens for the simultaneous detection of additional gastro-intestinal targets. Despite the current trend towards multiplex assays, single, duplex and triplex assays will remain useful in a research or public health settings when the investigation of a single pathogen is required and high sensitivity assay is needed.

**Limitations**: This study has some limitations. First, due to high sensitivity, molecular assays (triplex or Seegene assays) may detect more viruses than traditional methods. The clinical relevance of these findings may not be assured as some of these findings may be from asymptomatic patients. Specimens submitted to PHO laboratory for testing are usually collected from patients with clinical symptoms present; however, compliance with PHO laboratory testing guidelines cannot be assured. As well, patient’s specimens were not pre-treated with intercalating dye, such as ethidium monoazide (EMA) or propidium monoazide (PMA), prior to rRT-PCR to enables differentiation of infectious from non-infectious particles [34]. Second, the results of this study are based on a convenience set of specimens and not randomly selected specimens. In this study, the number of positive specimens for rotavirus or adenovirus was slightly higher than that reported in other studies [28,29,30]. This could also affect the performance indicators reported in this study. Last, specimens used in this study were retrospectively tested which may have affected the detection rate; however, since retrospective testing was performed for both triplex and Seegene assays the freeze-thaw effect should have affected both testing methods in a similar manner.

In conclusion, the laboratory–developed triplex assay provides a sensitive, specific, and efficient platform for the simultaneous detection of adenovirus and rotavirus in stool specimens among patients with gastroenteritis. Triplex assay detected more adenovirus and rotavirus than traditional methods. This assay can be effectively implemented in a clinical microbiology setting within and outside reference laboratories. The high performance and through-put, the lower cost than a commercial assay, the reduced TAT in addition to the potential for customizing the number of diarrheal viral targets, makes it a more desirable testing method. Pre-treatment of specimens with EMA or PMA (prior to rRT-PCR) in future studies, could eliminate some of the uncertainties about false positives and false negatives results and add more relevance to clinically usefulness. As such, it may lead to a lower burden of disease for hospital acquired gastrointestinal infections and a monetary saving for the institution for not keeping non-infectious patients in isolation.

## Figures and Tables

**Table 1:** Distribution of viral and bacterial pathogens used for validation of gastrointestinal triplex Assay.

Type	Pathogen	Number of Specimens	Source
Patients specimens	Adenovirus	39	Stool
	Rotavirus	349	Stool
	Negative specimens	297	Stool
Control specimens	*Samonella Paratyphi A*	2	Stool
	*Samonella Typhi*	1	Stool
	*Shigella Dysenteriae Type 2*	5	Stool
	*Escherichia coli - 0:157:H7*	1	Culture
	*Samonella enteritidis*	1	Culture
	*Shigella flexneri*	1	Culture
	*Shigella sonnei*	1	Culture
	*Vibrio cholera*	1	Culture
	*Camplyobacter jejuni*	1	Culture
	Influenza B	2	NP swabs
	Influenza A/ H1N1	5	NP swabs
	Influenza A/ H3N2	1	NP swabs
	Picornavirus	2	NP swabs
	*Aermonas hydrophila*	1	Culture
	*Neisseria meningitidis*	1	Culture
	*Streptococcus pyogenes*	2	Culture
	*Streptococcus pneumoniae*	2	Culture
	*Staphylococcus epidermidis*	1	Culture
	Total	716	

Footnote: Distribution of 685 and 32 stool and control specimens, respectively used for the development and validation of the triplex assay. Stool specimens were submitted for testing from February 2009 to February 2013.

**Table 2:** Primers and probes used for target amplification in the gastrointestinal virus triplex assay.

Target	Primers and Probe Sequences	Amp Size (BP)	NA Position	PCR Method	References
Adenovirus (hexon region)	FO: CAG GAC GCC TCG GRG TAY CTS AG RE: GGA GCC ACV GTG GGR TT PR: FAM-CCG GGT CTG GTG CAG TTT GCC CGC-BHQ	103	17649–17752 *	rRT-PCR	Damen, M. et al. 2008 [17]
Rotavirus (NSP3 gene)	FO: CCA TCT WCA CRT RAC CCT CTA TGA G RE: GGT CAC ATA ACG CCC CTA TAG C PR: CY5-AGT TAA AAG CTA ACA CTG TCA AA-BHQ	86	963–1049	rRT-PCR	Zeng, S. et al. 2008 [18]
*B. fragilis* (16S rRNA gene)	FO: GAG AGG AAG GTC CC RE: CGC TAC TTG GCT GG PR: FAM-CCA TTG ACC AAT ATT CCT CAC TGC TGC CT-BHQ	129	296–425	RT-PCR	Layton, A. et al. 2006 [24]
MS2 (MS2-TM2JOE)	FO: GGC TGC TCG CGG ATA CC RE: TGA GGG AAT GTG GGA ACC G PR: JOE-ACC TCG GGT TTC CGT CTT GCT CGT-BHQ1	201	3166–3367	rRT-PCR	Drier et al., 2005 [25]

Footnote: R, A/G; Y, C/T; V, A or C or G; W, A/T. FO, forward; RE, reverse; PR, probe; rRT-PCR, reverse real time reverse transcription PCR; Amp, amplicon; NA, nucleic acid. Base pair position for the adenovirus amplicon varies in different serotypes; * The position shown are for *hexon* gene of human adenovirus F41 strain (DQ#315364).

**Table 3:** Results obtained for adenovirus and rotavirus in the gastrointestinal triplex assay compared to Seegene assay, EM and ICT testing methods.

**A-Triplex Compared to Seegene**							
	Seegene result for Adenovirus				Seegene result for Rotavirus		
Triplex	Detected	Negative	Total	Triplex	Detected	Negative	Total
Adenovirus	43	7	50	Rotavirus	106	4	110
Negative	0	319	319	Negative	1	258	259
Total	43	326	369	Total	107	262	369
**B-Triplex Compared to EM**							
	EM result for Adenovirus				EM result for Rotavirus		
Triplex	Detected	Negative	Total	Triplex	Detected	Negative	Total
Adenovirus	37	23	60	Rotavirus	278	20	298
Negative	2	516	518	Negative	5	275	280
Total	39	539	578	Total	283	295	578
**C-Triplex Compared to ICT**							
	ICT result for Rotavirus						
Triplex	Detected		Negative		Total		
Rotavirus	145		23		168		
Negative	15		137		152		
Total	160		160		320		

**Footnote:** Distribution of testing results for adenovirus and rotavirus of specimens tested by two testing methods (triplex and Seegene), (triplex and EM), (triplex and ICT). PHO laboratory test: Seegene assay and EM detects adenovirus and rotavirus; ICT detects rotavirus.

**Table 4:** Measuring the accuracy of gastrointestinal triplex assay for identification of adenovirus and/or rotavirus against Seegene assay, EM and ICT testing methods.

**A-Performance of Triplex Assay Against a Reference Standard Method**					
			Seegene		
			Adenovirus		Rotavirus
Triplex Accuracy	Formula		Estimate % (95% CI)		Estimate % (95% CI)
Sensitivity	TP/(TP + FN) × 100		100 (91.7–100)		99.1 (94.9–99.8)
Specificity	TN/(FN + TN) × 100		97.8 (95.6–99.1)		98.4 (96.1–99.6)
PPV	TP/(TP + FN) × 100		86 (74.7–92.7)		96.3 (90.9–98.6)
NPV	TN/(FN + TN) × 100		100		99.6 (97.3–99.9)
**B-Performance of Triplex Assay Against Two Non-Reference Standard Methods**					
		EM			ICT
		Adenovirus		Rotavirus	Rotavirus
Triplex Accuracy	Formula	Estimate % (95% CI)		Estimate % (95% CI)	Estimate % (95% CI)
PPA	a/(a + c) × 100	94.8 (81.3–99.1)		98.2 (95.6–99.3)	90.6 (84.7–94.4)
NPA	d/(b + d) × 100	95.7 (93.5–97.2)		93.2 (89.5–95.7)	85.6 (79.0–90.4)
OPA	(a + d)/(a + b + c + d) × 100	95.6 (93.5–97.1)		95.6 (93.5–97.1)	88.1 (83.9–91.3)

**Footnote:** Sensitivity, Specificity, Positive Predictive Value and Negative Predictive Value were calculated when triplex was compared to a standard reference testing method. Positive Percent Agreement, Negative Percent Agreement, Overall Percent Agreement were calculated when triplex was compared to a non-standard reference testing method. TP-True positive, FN-false negative, TN-true negative, FN-false negative a-number of positive results concordant between both methods; b and c number of discordant positive/negative results between two methods, d- number of concordant negative results between two methods. For this comparison a performance below 85% was considered as ‘below average’, at 86%–90% as ‘average’ and above 90% as ‘above average’.

**Table 5:** A three-way comparison of results obtained for adenovirus and rotavirus in the gastrointestinal triplex assay compared to EM (A and B) or ICT (C) using Seegene assay as the standard reference method.

**A-Adenovirus Results**				
New Test	Non-Reference Standard Method	Total	Reference Standard Method	
			Seegene	
Triplex	EM		+	−
+	+	32	32	0
+	−	15	8	7
−	+	1	0	1
−	−	286	0	286
Total		334	40	294
**B-Rotavirus Results**				
New Test	Non-reference Standard Method	Total	Reference Standard method	
			Seegene	
Triplex	EM		+	−
+	+	97	97	0
+	−	7	3	4
−	+	2	0	2
−	−	228	0	228
Total		334	100	234
**C-Rotavirus Results**				
New Test	Non-Reference Standard Method	Total	Reference Standard Method	
Triplex	ICT		Seegene	
			+	−
+	+	46	46	0
+	−	7	5	2
−	+	6	1	5
−	−	85	0	85
Total		144	52	92

**Footnote:** In both analyses Seegene assay is the reference standard method. Results shown are per specimen based and are compared side to side. The sign under each test method indicates confirmed positive (+) or negative (−) results by each method. For this comparison a performance below 85% was considered as ‘below average’, at 86%–90% as ‘average’ and above 90% as ‘above average’.
